# The association of malocclusions with masseter muscle thickness based on ultrasonography measurements: a retrospective cohort study

**DOI:** 10.1093/ejo/cjaf084

**Published:** 2025-10-15

**Authors:** Dimitris Papagiannopoulos, Ioanna Georgiakaki, Maria Charalampidou, Anna-Bettina Haidich, Gregory S Antonarakis, Stavros Kiliaridis

**Affiliations:** Division of Orthodontics, University Clinics of Dental Medicine, University of Geneva, Rue Michel-Servet 1, Geneva 1206, Switzerland; Private Practice, Venloer Straße 107, Pulheim 50259, Germany; Division of Orthodontics, University Clinics of Dental Medicine, University of Geneva, Rue Michel-Servet 1, Geneva 1206, Switzerland; Department of Hygiene, Social-Preventive Medicine and Medical Statistics, Medical School, Aristotle University of Thessaloniki, Aristotle University of Thessaloniki University Campus, Thessaloniki 54124, Greece; Division of Orthodontics, University Clinics of Dental Medicine, University of Geneva, Rue Michel-Servet 1, Geneva 1206, Switzerland; Division of Orthodontics, University Clinics of Dental Medicine, University of Geneva, Rue Michel-Servet 1, Geneva 1206, Switzerland; Department of Orthodontics and Dentofacial Orthopedics, University of Bern, Freiburgstrasse 7, Bern 3010, Switzerland

**Keywords:** masseter muscle, ultrasound, malocclusions, deep bite, crossbite, BMI

## Abstract

**Aim:**

To investigate whether the functional capacity of elevator masticatory muscles, estimated by masseter muscle thickness, is associated with malocclusions in the sagittal, vertical, and transverse axes.

**Materials and methods:**

670 consecutive cases were evaluated. Pre-treatment dental casts were examined for malocclusion characteristics (overjet, sagittal molar relationships, overbite, posterior crossbite), and ultrasonographic measurements of the masseter muscles were used for thickness measurements. Gender, age, and BMI were also recorded. A multiple linear regression analysis was conducted to investigate the association of masseter thickness with age, gender, BMI, and malocclusions. A subgroup analysis across age groups explored whether age and gender had a different impact on their association with masseter muscle thickness, and a paired *t*-test examined possible asymmetry in masseter muscle thickness between the crossbite and non-crossbite sides.

**Results:**

Males showed thicker masseter muscles than females by 0.8 mm. Older-growing individuals presented thicker muscles, with a 1-year age increase resulting in a 0.1 mm increase in mean thickness, and patients with increased values of BMI presented thicker muscles. A 1mm overbite increase was associated with 0.1mm thicker masseter muscle. Patients with unilateral posterior crossbite showed thinner muscles on their crossbite (11.15 mm) than the non-crossbite side (11.42 mm) with a mean difference of 0.27 mm (*P* = 0.002).

**Conclusions:**

Masseter muscle thickness was greater in males, subjects with increased values of BMI, and older-growing individuals, showing age-related growth that plateaued in adulthood. Patients with deep bite had thicker muscles, while patients with unilateral posterior crossbite showed thinner muscles on their crossbite side than on the non-crossbite side.

## Introduction

Clinical and experimental studies have shown that the functional capacity of the masticatory muscles may influence dentofacial morphology [1–5]. The importance of these muscles can be further seen in orthodontics with their impact on treatment outcomes and stability [6, 7]. A large inter-individual variation in the dentoalveolar changes across growing individuals treated with functional appliances for Class II malocclusion has demonstrated that children with thinner pre-treatment masseter muscles present greater dentoalveolar changes towards Class I sagittal relationships [6], while in patients undergoing orthognathic surgery for Class III malocclusion, greater masticatory muscle activity, as measured by electromyography, has been associated with a higher rate of post-surgical relapse [7].

Given the significance of the masticatory muscles in dentofacial morphology, treatment outcomes, and stability, further investigations are warranted to better understand the importance of their functional capacity. In this regard, the masseter muscle lends itself to experimental investigation as it has been demonstrated to serve as a representative muscle, reflecting the functional capacity of all the masticatory elevator muscles through its thickness [8].

Different methods for evaluating the relationships between craniofacial characteristics and functional muscular capacity have been used, either by recording the maximal bite force [9–11] or by measuring the muscle thickness using techniques such as ultrasound scanning [12, 13], computerized tomography (CT) [14], or magnetic resonance imaging (MRI) [15, 16]. The superficial location of the masseter muscle facilitates easy implementation of quantitative measurements of its cross-sectional thickness, including those conducted through ultrasonography, which provides a rapid, reliable, simple, and economical approach for precise measurements.

The vast majority of the studies have previously investigated the associations between masseter muscle thickness and craniofacial morphology by looking into skeletal discrepancies [17–23]. Few studies seem to investigate the association between masseter muscle thickness and dental malocclusions, (Meher *et al*. (2024) [24] and Kaya *et al*. (2025) [25]) that focused on age groups from 16 to 26 years and from 11 to 15 years of age, respectively.

Concerning the fact that numerous parameters may influence the thickness of the masseter muscle, it would be appropriate to have a substantial sample size for the statistical analysis and for the covariates to be compared appropriately.

Nevertheless, large-scale studies evaluating the association between the capacity of the masticatory muscles and malocclusions occurring in the sagittal, vertical, and transverse planes in both growing and adult individuals are missing.

The present study aims to associate malocclusions in the sagittal, vertical, and transversal axes with the thickness of the masseter muscle, measured using ultrasonography, in a sample of pre-orthodontic patients. The null hypothesis was that there is no association between malocclusions and masseter muscle thickness.

## Materials and methods

### Materials

The present study was approved by the Cantonal Commission for Research Ethics (no. 2024-00195). The investigation was based on a total of 1150 files of patients treated in a single orthodontic clinic between 1996 and 2015, a period during which ultrasound measurements of the masseter muscles of patients had taken place. 480 cases did not have ultrasound measurements and thus were excluded from the study, with 670 consecutive cases remaining.

The inclusion criteria were: Pre-orthodontic patients regardless of sex, age, and malocclusion, and complete orthodontic pre-treatment records including intact dental casts and ultrasonographic measurements of masseter muscle thickness bilaterally.

Exclusion criteria were: patients with missing or incomplete records; patients with craniofacial anomalies, including cleft lip and palate; patients with temporomandibular pathology or dysfunction.

In the included cases, gender and age were recorded. Subjects were also subdivided into three age groups, namely: ‘children’ (4 to 12 years), which was the reference group, ‘adolescents’ (>12 to <18 years), and ‘adults’ (≥18 to 34 years).

The malocclusions were evaluated on pre-treatment dental casts, and ultrasonographic measurements of the masseter muscles were used for muscle thickness measurements.

### Methods

#### Malocclusions on dental casts

Several measurements were made on the pre-treatment dental casts. In the anteroposterior axis, overjet was measured in millimetres from the labial surface of the mandibular incisors to the labial surface of the maxillary incisors with the teeth in centric occlusion. Dental sagittal relationships were recorded based on Angle’s classification, with Class I attributed to those patients having Class I to ¼-cusp towards Class II or Class III molar relationships. Class II cases were described as those having more than a ¼-cusp to full-cusp Class II molar relationships, while Class III cases were defined as those having molar relationships of more than a ¼-cusp to full-cusp Class III [26].

On the vertical axis, overbite was measured in millimetres from the incisal edge of the mandibular incisors to the incisal edge of the maxillary incisors with the teeth in centric occlusion. On the transverse axis, posterior crossbite was attributed to those patients having two or more posterior teeth in crossbite. The side of the crossbite was also recorded as was its unilateral or bilateral nature.

#### Masseter muscle thickness

**Figure 1.**
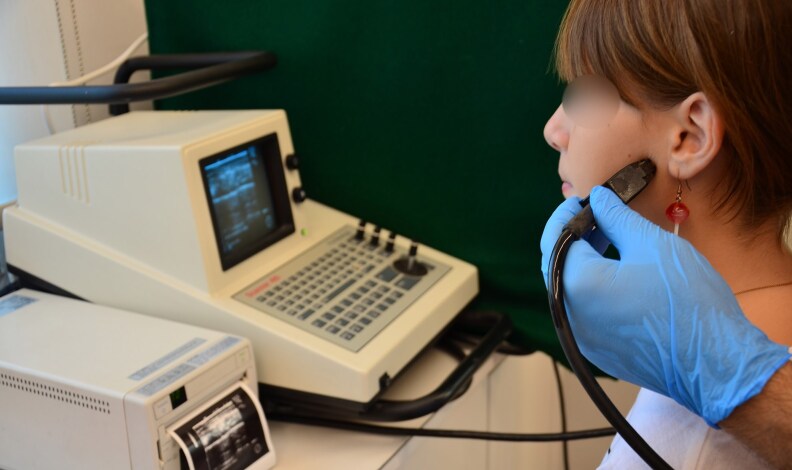
Ultrasound examination of the masseter muscle. A linear-array transducer (7.5 MHz) connected to a Pie Medical Scanner 480 was positioned over the masseter region, with the participant seated upright and the head maintained in a natural posture.

**Figure 2.**
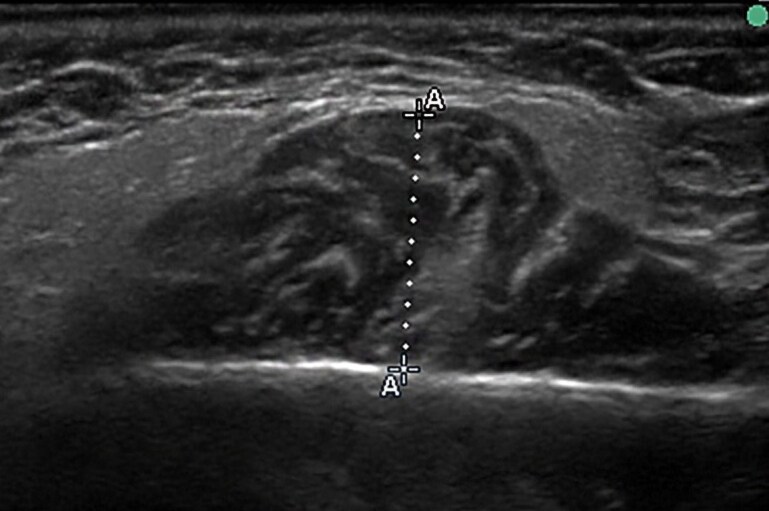
Transverse ultrasonographic image of the masseter muscle. The skin and subcutaneous tissue lie above the superficial fascia (thin echogenic line). The masseter muscle appears hypoechoic with internal striations beneath the fascia. The lateral surface of the mandibular ramus is visible as a bright echogenic contour with posterior acoustic shadowing. Electronic calipers measure the distance between the superficial fascia and the mandibular surface to assess muscle thickness, as is shown with the letter A in the figure.

The thickness of the masseter muscle was measured according to the method proposed by Kiliaridis & Kälebo and modified by Raadsheer *et al*. [12, 27], as part of the routine pre-orthodontic examination. Measurements were performed by the same operator (IG) after being calibrated with the senior author. Ultrasound images were acquired using a real-time scanner (Pie Medical Scanner 480) equipped with a 7.5 MHz linear-array transducer. Participants were positioned upright, with their heads in a natural position, without the use of a headrest. The masseter muscles were scanned bilaterally on a level halfway between the zygomatic arch and the gonial angle (Fig. 1). Thickness was defined as the maximum perpendicular distance between the ramus and the superficial muscle surface. The transducer was gently pressed against the cheek using a rich amount of gel, and oriented perpendicularly to the ramus cortex, since oblique scanning would increase the muscle thickness values. Adjustments were made to ensure the correct inclination of the transducer until the ramus appeared as a distinct white line on the screen. Participants alternately clenched and relaxed their jaws to enhance contrast between muscle and subcutaneous tissue (Fig. 2).

Measurements were taken during muscle contraction in maximal intercuspal clenching, as well as during muscle relaxation.

The random error of the method of this experienced operator, for the measurements performed under maximal contraction, calculated after a one-month interval, was very small, 0.2 mm, while for the measurements performed under muscle relaxation was 0.3 mm [28].

The random error of the method was minimized through operator calibration, long period of experience, and adherence to a standardized measurement protocol where each muscle was imaged twice per side, with some interval to prevent muscle fatigue. The thickness per side was calculated as the mean of the two measurements, and overall masticatory muscle function was estimated as the average of the masseter thickness of both sides.

#### Statistical analysis

Continuous variables were presented with means and standard deviations (SD) while categorical variables with frequencies and percentages.

For patients with unilateral posterior crossbite, a paired *t*-test was performed after ascertainment of normality. The same analysis was used to explore the differences in masseter muscle thickness between the crossbite and non-crossbite sides, as well as the left and right sides for patients with bilateral crossbite and patients without any transverse malocclusion.

Simple linear regression models were carried out to investigate the association between masseter muscle thickness and variables that were considered clinically relevant, such as age, gender, BMI, overjet, sagittal molar relationships (using Angle’s classification), transverse malocclusions, and overbite. All variables with *P* < 0.20 in univariate analysis were included in the multiple linear regression models with masseter muscle thickness as the dependent variable, either in maximal contracted state or in muscle relaxation state. Additionally, a subgroup analysis by different age groups was performed using a multiple linear regression model to investigate whether age and gender of the patients had a different impact on their association with masseter muscle thickness.

The overall masticatory function for contraction and relaxation states (average of the 4 measurements) was used in the multiple linear regression analyses, while for the comparison between the crossbite and non-crossbite sides, the average of the two measurements on each side was used.

Beta coefficients with the corresponding 95% confidence intervals (CI) were presented. All statistical analyses had a significance level of 0.05 and were performed using R Studio software version 4.2.1.

## Results

### Description of the material

The present study involved 670 patients, with the majority being females (59.4%) and the mean age was 11.8 years (SD = 4.4 years). Among these subjects, most were children (66.4%), with a mean age of 9.6 years; approximately a quarter were adolescents (25.8%), with a mean age of 13.8 years; and a smaller proportion were adults (7.7%), with a mean age of 24.2 years. The description of the material is depicted in Table 1.

**Table 1.** Description of the material.

Characteristics	*n* = 670
Gender, *n* (%)	
Males	272 (40.6)
Females	398 (59.4)
Mean age, years (SD)	11.8 (4.4)
Age groups, *n* (%)	
Children	445 (66.4)
Adolescents	173 (25.8)
Adults	52 (7.7)
Mean BMI, (SD)	19.6 (3.6)
Mean overjet, mm (SD)	4.2 (3.2)
Mean overbite, mm (SD)	3.0 (2.2)
Angle Classification, *n* (%)	
Class I	274 (40.9)
Class II	348 (51.9)
Class III	48 (7.2)
Transverse relationships, *n* (%)	
Non-crossbite cases	543 (81.0)
Unilateral crossbite cases	80 (11.9)
Bilateral crossbite cases	47 (7.0)
Mean masseter muscle thickness under contraction, mm (SD)	11.6 (1.6)
Mean masseter muscle thickness under relaxation, mm (SD)	11.5 (1.5)

Continuous variables are presented with means and standard deviations (SD), while categorical variables are presented with frequencies and percentages.

### Masseter muscle thickness and transverse malocclusions

In the unilateral posterior crossbite cases, the mean masseter muscle thickness for the non-affected side (11.42 mm) was thicker than the affected side (11.15 mm) with a mean difference of 0.27 mm (*P* = 0.002). No differences were found between the right (11.37 mm) and left (11.53 mm) sides in bilateral crossbite cases, with a mean difference of 0.16 mm (*P* = 0.13) as well as between the right (11.65 mm) and left (11.70 mm) sides in the subjects without crossbite, with a mean difference of 0.05 (*P* = 0.09).

### Factors associated with masseter muscle thickness

We performed the statistical analysis with the dependent variable being the masseter muscle thickness, both in maximal contraction state and in relaxation state. The results were very similar, with the two variables of masseter muscle (under contraction and under relaxation) sharing a very high correlation coefficient (*r* = 0.97, *P* ≤ 0.001). As the error method for the masseter muscle thickness at maximal contraction was smaller (0.2 mm) compared with the muscle thickness under relaxation (0.3 mm), only the results from the masseter muscle measured under maximal contraction will be presented. (The rest of the results are presented with the help of Supplementary Table S1 in the online version).

In univariate analyses, gender, age, BMI, overjet, overbite, sagittal dimension, and transverse dimension were associated with masseter muscle thickness. However, when all variables were included in a multiple regression model, gender, age, BMI, and the malocclusions of the vertical dimension were found to be associated with the masseter muscle thickness.

Males showed thicker masseter muscles by 0.8 mm compared with females after adjustment for the other variables.

For a 1-year increase in the age of the patients, the mean masseter muscle thickness increased by 0.1 mm, for each 1-point increase in BMI, the mean masseter muscle thickness increased by 0.14 mm, and for each 1 mm increase in the overbite, there was an increase in the mean masseter muscle thickness by 0.1 mm after adjustment for the other variables.

The results of the simple and multiple linear regression models are depicted in Table 2.

**Table 2.** Factors associated with masseter muscle thickness in contraction.

	Univariate analysis			Multivariate analysis		
Variables	Unadjusted β	95% CI	*P*-value	Adjusted β	95% CI	*P*-value
Gender (males vs females)	0.46	0.21, 0.71	<0.001	0.8	0.35, 1.26	<0.001
Age (years)	0.15	0.12, 0.17	<0.001	0.1	0.04, 0.15	<0.001
BMI	0.19	0.13, 0.26	<0.001	0.14	0.07, 0.2	<0.001
Overjet (mm)	0.04	0.007, 0.08	0.02	0.02	−0.06, 0.11	0.563
Overbite (mm)	0.17	0.11, 0.23	<0.001	0.1	−0.002, 0.21	0.055
Class I Angle	Reference			Reference		
Class II Angle	0.14	−0.11, 0.4	0.26	−0.15	−0.72, 0.41	0.598
Class III Angle	−0.68	−1.19, −0.16	0.009	−0.41	−1.35, 0.52	0.386
Non-crossbite cases	Reference			Reference		
Unilateral crossbite cases	−0.38	−0.77, 0.002	0.05	0.09	−0.66, 0.85	0.805
Bilateral crossbite cases	−0.19	−0.69, 0.31	0.45	−0.22	−1.02, 0.57	0.580

The beta coefficients, 95% CIs, and the *P*-values are depicted for each variable.

For the Multivariate model: *R*² = 0.3, *P* value = <0.001.

### Masseter muscle thickness with age and gender

Male children presented 0.35 mm thicker masseter muscles compared with female children (*P* = 0.005), whereas in adolescence and adulthood, males showed thicker masseter by 1.29 mm (*P* < 0.001) and 1.81 mm (*P* = 0.002), respectively, compared with women. Regarding age, for a 1-year increase in the age of the patients during childhood and adolescence, the mean masseter muscle thickness increased by 0.23 mm and 0.24 mm, respectively (*P* < 0.001 for both age groups), whereas during adulthood, the mean masseter muscle thickness showed minimal change with the increase in age (*P* = 0.732). The results of the multiple regression model are depicted in Table 3.

**Table 3.** Association of masseter muscle thickness with age and gender of the patients in different age groups.

	Children (*n* = 445)			Adolescents (*n* = 173)			Adults (*n* = 52)		
Variables	Adjusted β	95% CI	*P*-value	Adjusted β	95% CI	*P*-value	Adjusted β	95% CI	*P*-value
Gender (males vs females)	0.35	0.10, 0.60	<0.001	1.29	0.8, 1.79	<0.001	1.81	0.7, 2.91	0.001
Age (years)	0.23	0.14, 0.31	<0.001	0.24	0.06, 0.41	<0.001	0.01	−0.08, 0.12	0.732

The beta coefficients, 95% CIs, and the *P*-values are depicted for each variable.

## Discussion

The results of the present study reject the null hypothesis, indicating an association between malocclusions and masseter muscle thickness. Males, older-growing individuals as well as individuals with higher values of BMI, manifested thicker masseter muscles. Increased overbite was also associated with thicker masseter muscles. In the transverse dimension, patients with unilateral posterior crossbite had thinner masseter muscles on their crossbite side than on the contralateral non-crossbite side, unlike patients with bilateral crossbite and without transverse malocclusion, where muscle thickness was almost symmetrical. In the univariate analyses, overjet and sagittal malocclusions (using Angle’s classification) showed an association with masseter muscle thickness as well.

Nevertheless, these associations lost significance when the vertical dimension was included in the multiple regression model, suggesting an interaction between overbite and malocclusions of the sagittal and transverse axes, that increased multicollinearity and weakened the association of masseter muscle thickness with overjet, Angle classification, and unilateral crossbite variables. Therefore, the results of the multiple regression analysis are those emphasized in the present study.

Several other studies have assessed the functional capacity of the masticatory muscles by exploring its association with dentofacial morphology through maximal bite force [9, 10, 29] or by measuring masseter thickness with CT [14], MRI [15, 16] or ultrasonography [12], which was found to be a reliable and accurate method.

In our study, we presented the measurements performed only under maximal clenching since relaxed muscles permit greater probe compression on the cheek, thus, increasing the measurement error [12].

Regarding the transverse axis, the mean difference found in masseter muscle thickness between the affected and non-affected sides in patients with unilateral posterior crossbite does not lead to any clinically -or aesthetically- noticeable asymmetry, and thus no direct clinical benefits exist from the present findings for the individual patient. Nevertheless, it does provide support to the assumption of an asymmetric function of the masticatory muscles. Our results align with those of Kiliaridis *et al*. [30], who found in patients with unilateral functional crossbite that the thickness of the masseter muscles on the crossbite side was statistically significantly thinner than on the normal side, likely due to asymmetric muscle activity adapting to avoid cuspal interference. Our sample did not allow for the identification of individuals with posterior crossbite with or without functional shift, and they were thus all pooled together. Nevertheless, it can be considered that most patients in our study had a functional mandibular shift as is the case in epidemiological studies [31].

Our findings in growing individuals align with cephalometric studies on adults concerning the association between masseter muscle thickness and vertical facial height [12, 17, 18, 32, 33], as malocclusions often correlate with the underlying skeletal morphology.

The aforementioned studies found a negative correlation between masseter muscle thickness and vertical facial height, where thicker muscles were associated with brachycephalic skulls, short face morphology, and decreased intermaxillary and gonial angles. Such alterations can be explained by the stimulation of osteoblastic activity and the addition of bone matrix at the periosteal surface of the region of masseter muscle attachment, thus resulting in a more acute gonial angle [34].

Conversely, thin masseter muscles in long-face patients may allow excessive eruption of posterior teeth and eventually cause backward mandibular rotation as continued eruption is influenced by masseter muscle thickness [35].

Thus, the observed association we found in the present study between increased overbite and masseter hypertrophy could be explained by the hypothesis that masticatory muscles do influence dentoalveolar adaptation, as demonstrated in experimental models with botulinum toxin-induced masseter hypotrophy in growing rats, which showed compensatory dentoalveolar changes—such as molar supraeruption—in response to altered muscle activity [36].

Similarly, daily chewing exercise therapy with a tough chewing gum material that was instituted in 13 children for a year showed a significant increase in the bite force and muscle activity during maximal bite, followed by a significant increase in the overbite of those children [37].

Nevertheless, the observed association between increased overbite and masseter hypertrophy can be explained by other hypotheses, too; The presence of certain malocclusions, *per se*, may influence the functional condition of the masticatory muscles, as in cases with functional posterior crossbite, where there is an asymmetric thickness of the masseter muscles [30], a finding that is also confirmed in the present study.

This relationship is further supported by evidence demonstrating that such muscular asymmetry tends to resolve following correction of the malocclusion [38].

Another explanation could be that bad oral habits may also influence the functional condition and, ultimately, the thickness of the masseter muscle, as they may affect the position of the teeth, masticatory muscles, and temporomandibular joints, thus disturbing stomatognathic function or worsening pre-existing disorders [39–42].

Bad oral habits, such as mouth breathing, can ultimately have an impact on the masseter muscle. Neves-Leal *et al*. 2024 found using electromyography that oral breathers exhibited lower electrical muscle activity of the masseters during chewing compared with the nasal breathers [43].

Existence and persistence of oral habits may interfere with children’s dentofacial growth, leading to malocclusions and skeletal discrepancies. Therefore, both the presence of malocclusions and changes in masseter muscle characteristics may share a common aetiological background rooted in persistent aberrant oral behaviours [44].

Regarding age, our results showed that older-growing individuals presented thicker muscles. This agrees with the results of Newton *et al*. [45], who found a strong correlation between age and masseter muscle thickness, and Tentolouri *et al*. [19], who found that older children present thicker masseter muscles. When additional multiple regression models were carried out to investigate the association of aging with masseter muscle thickness across the different age groups, it was found that the rate of masseter muscle growth differs from childhood and adolescence to adulthood, where aging is no longer associated with a further increase in muscle thickness. This is consistent with Lexell *et al*. [46], who found in cross sections of autopsied vastus lateralis muscles a progressive increase in muscle volume from childhood to adulthood with the maximal muscle area found in the mid-twenties, as well as Lexell *et al*., [47] who found that there is age-related atrophy beginning approximately at 25 years of age [47].

Concerning gender, male patients showed thicker masseter muscles than females. The sex differences in muscle thickness were more prominent as the patients got older, with the highest difference in muscle thickness seen during the adulthood period. Such results are aligned with general muscle physiology and the results of Charalampidou *et al*. [28], who found that young males had thicker masseter muscles than young females in both the relaxed and contracted conditions. The etiologic factor for such differences across the two genders may be attributed to the different sex hormones that can influence the masseter muscle fibre-type composition [1]. A strong point of the study is its large sample size (*n* = 670), making it one of the largest studies examining associations between masseter muscle thickness and malocclusions. The sample was drawn from the same centre, ensuring no differences across the population or the operator carrying out the ultrasonographic measurements. However, since the sample was drawn from an orthodontic clinic, all participants had some degree of malocclusion. While some had minor discrepancies, most presented moderate to severe malocclusions, which limit the generalizability of these findings to a healthy population with normal occlusion.

Clinical significance: Understanding that certain malocclusions are linked to the functional capacity of the masticatory muscles can aid both in diagnosis as well as in treatment planning and prognosis of long-term treatment outcomes and stability.

More specifically, malocclusions of the vertical and transversal dimensions could get in the future a better differential diagnosis based not only on the subject’s pure dental and skeletal phenotype, but also on the individual’s functional capacity.

## Conclusion

- Patients with unilateral posterior crossbite show significantly thinner masseter muscles on the crossbite side than on the non-crossbite side.
- Masseter muscle thickness was greater in males, subjects with increased values of BMI, and older-growing individuals, showing age-related growth that plateaued in adulthood.
- In the vertical axis, patients with thicker masseter muscles presented bigger vertical overbite.
- Masseter muscle thickness was not associated with malocclusions in the sagittal plane.

## Supplementary Material

cjaf084_Supplementary_Data

## Data Availability

The datasets used and analysed during the current study are available from the corresponding author upon reasonable request.
