# Supplementary material for: The association of malocclusions with masseter muscle thickness based on ultrasonography measurements: a retrospective cohort study
Source: Eur J Orthod. 2025 Oct 15;47(5):cjaf084. doi: 10.1093/ejo/cjaf084 (PMC12527289; doi:10.1093/ejo/cjaf084)
Supplement: cjaf084_Supplementary_Data [file cjaf084_supplementary_data.docx]

Supplementary material Table 1: Factors associated with masseter muscle thickness in relaxation

|  | Univariate Analysis | | | Multivariate Analysis | | |
| --- | --- | --- | --- | --- | --- | --- |
| Variables | Unadjusted β | 95% CI | p-value | Adjusted β | 95% CI | p-value |
| Gender (males vs females) | 0.53 | 0.23, 0.83 | < 0.001 | 0.77 | 0.32, 1.22 | < 0.001 |
| Age (years) | 0.11 | 0.08, 0.14 | < 0.001 | 0.09 | 0.04, 0.14 | < 0.001 |
| BMI | 0.18 | 0.12, 0.24 | < 0.001 | 0.12 | 0.06, 0.19 | < 0.001 |
| Overjet (mm) | -0.005 | -0.05, 0.04 | 0.832 | 0.02 | -0.07, 0.11 | 0.655 |
| Overbite (mm) | 0.1 | 0.04, 0.17 | 0.001 | 0.09 | -0.06, 0.20 | 0.065 |
| Class I Angle | reference reference | | | | | |
| Class II Angle | -0.003 | -0.3, 0.3 | 0.984 | 0.24 | -0.81, 0.32 | 0.401 |
| Class III Angle | -0.29 | -1, 0.4 | 0.423 | -0.36 | -1.33, 0.6 | 0.458 |
| Non-crossbite cases | reference reference | | | | | |
| Unilateral crossbite cases | -0.12 | -0.58, 0.34 | 0.611 | 0.19 | -0.56, 0.95 | 0.609 |
| Bilateral crossbite cases | -0.01 | -0.64, 0.6 | 0.957 | - 0.06 | -0.86, 0.73 | 0.864 |
| For the Multivariate model: R² = 0.28, p value = < 0.001 | | | | | | |

Supplementary material Table 1: The beta coefficients, 95% CIs, and the p-values are depicted for each variable.
